# 153. Sensitivity of MRSA Nasal Screening in Patients with *Staphylococcus aureus* Pneumonia: Impact of a More Sensitive Comparator

**DOI:** 10.1093/ofid/ofad500.226

**Published:** 2023-11-27

**Authors:** Irene-Constantina Papamanolis, Arnold Decano, ioannis Zacharioudakis, Yanina Dubrovskaya, Justin Siegfried, Kassandra Marsh, Dana Mazo, Kenneth Inglima

**Affiliations:** NYU Langone Hospital - Brooklyn, Brooklyn, New York; NYU Langone Health, New York City, New York; New York University, New York city, New York; NYU Langone Health, New York City, New York; NYU Langone Health, New York City, New York; NYU Langone Health, New York City, New York; New York University, New York city, New York; NYU Langone Health, New York City, New York

## Abstract

**Background:**

*Staphylococcus aureus* (*S. aureus*) pneumonia (PNA) is associated with significant morbidity and mortality. *Methicillin-resistant Staphylococcus aureus* (MRSA) nasal screening has been considered a valuable tool in guiding the de-escalation of antibiotics, particularly vancomycin, among patients with PNA by identifying patients with MRSA colonization in light of its high sensitivity and negative predictive value in earlier studies. However, the performance of MRSA/MSSA nasal screening has yet to be re-evaluated after more sensitive molecular methods for microbiologic diagnosis of PNA have become available. This study aims to determine the sensitivity of MRSA/MSSA nasal screening in patients diagnosed with *S. aureus* PNA based on the BIOFIRE® FILMARRAY® Pneumonia Panel results.

**Methods:**

We conducted a retrospective cohort study involving 346 patients with a positive BIOFIRE® FILMARRAY® Pneumonia Panel for MRSA or MSSA. Inclusion criteria were age >18 and use of MRSA/MSSA nasal screening by the culture within 48 hours of symptom onset. Exclusion criteria included polymicrobial PNA detected by the Pneumonia panel, infection(s) at other sites and known recurrent *S. aureus* infections.

**Results:**

A total of 56 patients with *S. aureus* PNA met the inclusion criteria. Of the 56 patients, 22 had mecA/C or MREJ detected, indicating MRSA infection. Among these 22 patients with MRSA PNA, 11 (sensitivity 50%) were screened positive by nasal culture. Among the 34 patients with MSSA PNA, 16 screened positive by nasal culture (sensitivity of 47.1%).

**Conclusion:**

The MRSA nasal screening culture demonstrated a sensitivity of 50% in patients with MRSA PNA, which is lower than what has been reported, including those using PCR. When using culture screening methods, risk factors for MRSA PNA and clinical and radiographic features will need to be considered for empiric antimicrobial therapy pending microbiologic test results. Future studies should focus on refining MRSA screening methodologies, evaluating their clinical utility in managing empiric antimicrobial therapy in patients with pneumonia, and studying the clinical outcomes in patients who received inappropriate therapy based on negative MRSA screening results.

**Disclosures:**

**All Authors**: No reported disclosures

